# Phenobarbital for the management of severe acute alcohol withdrawal (the PHENOMANAL trial): a pilot randomized controlled trial

**DOI:** 10.1186/s40814-021-00963-4

**Published:** 2022-01-22

**Authors:** Niall Filewod, Stephen Hwang, Christian J. Turner, Leena Rizvi, Sara Gray, Michelle Klaiman, Danielle Buell, Johnathan Ailon, Alexander Caudarella, Galo F. Ginocchio, Marlene Santos, Gyan Sandhu, Norman Dewhurst, Kelly Sequeira, Karen E. A. Burns

**Affiliations:** 1 Trillium Health Partners, Mississauga, Ontario Canada; 2 Unity Health Toronto – St. Michael’s Hospital, Toronto, Ontario Canada; 3 Divisions of Critical Care, Internal Medicine, Emergency Medicine, Psychiatry, Addictions, Family Medicine, and Pharmacy, University of Toronto, Toronto, Ontario Canada; 4 Li Ka Shing Knowledge Institute, Toronto, Ontario Canada; 5 Map Centre for Urban Health Solutions, Toronto, Canada; 6 University of British Columbia, Vancouver, British Columbia Canada

**Keywords:** Phenobarbital; Alcohol withdrawal, Delirium tremens, Treatment

## Abstract

**Background:**

Benzodiazepines are considered first-line treatment for patients experiencing severe acute alcohol withdrawal syndrome (sAAWS). Although several medications have been evaluated as potential adjuvant treatments for sAAWS, barbiturates show particular promise.

**Objective:**

In the PHENOMANAL trial, we will assess the feasibility of conducting an allocation-concealed, quadruple-blinded, randomized controlled trial (RCT) comparing symptom-triggered benzodiazepine therapy with either a single dose of adjuvant intravenous (IV) phenobarbital (7.5 mg/kg of ideal body weight) or a single dose of matching IV placebo for patients with sAAWS.

**Methods:**

We will recruit adult patients from the Emergency Department, Intensive Care Unit, or hospital wards with a Clinical Institute of Withdrawal – Adult revised (CIWA-Ar) score of 16 or more after receipt of at least 60 mg of diazepam or equivalent within 16 h of diagnosis of sAAWS, and an anticipated need for hospitalization. We will randomize participants (*n*=39) in a 2:1 manner to treatment and placebo groups, respectively. The primary objective of the PHENOMANAL pilot trial will be to demonstrate our ability to recruit the desired population over the trial period. As secondary objectives, we will evaluate clinician compliance with the treatment protocols, assess crossover rates from the placebo arm to the treatment arm, and obtain preliminary estimates of treatment effect. All trial participants will be followed for 7 days or until hospital discharge.

**Relevance:**

The PHENOMANAL trial is novel in investigating a new treatment for a common and understudied condition, repurposing an existing medication for a novel indication, and addressing an important evidence gap. Through conduct of the multidisciplinary pilot trial, we aim to advance methodology in acute care research through the use of a hybrid consent model and inform the design of a large-scale trial.

**Trial registration:**

ClinicalTrials.gov Registration NCT03586089; first registered July 13, 2018.

## Introduction

Approximately 20,000 Canadians are hospitalized each year for alcohol-related conditions at a cost of 150 million CAD/year [1]. Additionally, up to 25% of patients who are hospitalized for surgery or common medical conditions experience alcohol withdrawal [2–4]. At present, benzodiazepines are considered first-line treatment for patients experiencing severe acute alcohol withdrawal syndrome (sAAWS) [5–7]. Although several medications have been evaluated as potential adjuvant treatments for sAAWS in meta-analyses [8–11], barbiturates have been highlighted as a promising treatment in four clinical practice guidelines [12–15] due to their benzodiazepine-sparing effect and excellent safety profile. In a small RCT, Rosenson and coworkers found that a single dose of IV phenobarbital (10 mg/kg), in addition to lorazepam, vs. lorazepam alone, significantly decreased the rate of admission to the ICU (25% to 8%) and the need for a lorazepam infusion (31% to 4%), with no increase in complications [16]. However, this trial was limited to patients presenting to the emergency department (ED) with sAAWS, investigated symptom-triggered lorazepam treatment (not the current standard of care), and only reported on 50% of randomized participants for various reasons including an inability to obtain first-party consent [16].

Although benzodiazepines are commonly used to treat patients suffering from sAAWS, they have only been shown to be superior to placebo. No data support the superiority of benzodiazepines over other medications. Benzodiazepines are not an ideal treatment for sAAWS due to their short half-life and the requirement for repeated dosing in response to symptoms and signs of withdrawal [5, 12–15]. Moreover, some patients with sAAWS are benzodiazepine-resistant [17]. Consequently, no evidence exists to inform clinicians’ choice of second-line treatments for patients with sAAWS.

Phenobarbital is an inexpensive and widely available medication that is used to treat seizures. Phenobarbital may be an ideal treatment for sAAWS as it has an affinity for the GABA receptor (which is modulated by both alcohol and benzodiazepines), has a rapid time-to-peak, and is slow to taper. A single dose of intravenous (IV) phenobarbital given early during alcohol withdrawal is expected to reduce the need for symptom-triggered benzodiazepine administration as well as the development of complications related to sAAWS (e.g., seizures, aspiration, dysrhythmias).

### Trial overview

The PHENObarbital for the MANagement of severe ALcohol withdrawal (PHENOMANAL) trial is an allocation-concealed, quadruple-blinded, placebo-controlled pilot randomized controlled trial (RCT) comparing symptom-triggered benzodiazepine therapy with either a single dose of adjuvant intravenous (IV) phenobarbital or a single dose of matching IV placebo for patients with sAAWS.

### Objectives

The primary objective of the PHENOMANAL trial will be to demonstrate our ability to recruit 39 patients over the trial period. As secondary objectives, we will (i) evaluate clinician compliance with the treatment protocols, (ii) assess crossover rates from the placebo arm to the treatment arm, and (iii) obtain preliminary estimates of treatment effect on outcomes [cumulative benzodiazepine dose administered, rates of unanticipated admissions to ICU and/or monitored-care settings, adverse and serious adverse events (aspiration, intubation, new seizure, new dysrhythmia), hospital length of stay (LOS), time until “Clinical Institute for Withdrawal Assessment Adult revised” (CIWA-Ar) score is 10 or less on three consecutive occasions, time to treatment discontinuation and to addictions medicine consult, and vital status at hospital discharge] [18].

## Methods

We will include patients who meet all of the following criteria:

1. Alcohol withdrawal syndrome (regardless of admitting diagnosis).
2. Severe symptoms, as defined by a “Clinical Institute for Withdrawal Assessment Adult revised” (CIWA-Ar) score of 16 or more [6, 18], or delirium severe enough to prohibit assessment with the CIWA-Ar, or observed withdrawal seizures, in each case despite treatment with at least 60 mg of diazepam (or an equivalent dose of another benzodiazepine) in the previous 16 h, regardless of route of administration.
3. Early alcohol withdrawal, defined as the first 16 h after the diagnosis of acute alcohol withdrawal has been made. The time of diagnosis will be taken to be the time of prescription of symptom-triggered benzodiazepine therapy (“CIWA protocol”), or the time of consultation to ICU/Emergency Department, Internal Medicine, or the Addictions Service (Psychiatry) for the management of alcohol withdrawal.
4. Anticipated need for hospitalization (i.e., admission to ICU, medical step-up unit, or wards).

We depict trial exclusion criteria in Table 1.

**Table 1:** Exclusion criteria

(i) An alternate etiology for delirium thought to be more likely than alcohol withdrawal	
(ii) Age <16 years	
(iii) Pregnancy (positive assay for ßhCG—a urine assay or blood test will be performed for all women < 55 years)	
(iv) Current breastfeeding	
(v) Severe acute hepatitis (AST or ALT >500); liver failure (INR >2 not otherwise explained)	
(vi) A presenting complaint of neurotrauma, brain mass, or intra-cranial bleed; abnormal cell count or gram stain on lumbar puncture (if performed)	
(vii) A strong clinical suspicion of recent co-ingestion of depressant drugs (e.g., opioids, toxic alcohols, gamma-hydroxy-butyrate)	
(viii) Hemodynamic instability (systolic blood pressure [SBP] < 90 mmHg)	
(ix) History of barbiturate allergy	
(x) History of porphyria	
(xi) History of myasthenia gravis	
(xii) Inability to obtain IV access	
(xiii) Anticipated transfer to another center	
(ivx) Stated intent to leave against medical advice	
(xv) Active outpatient prescription for anti-retroviral therapy for HIV	
(xvi) Active outpatient prescription for one of the following anti-epileptic drugs: valproic acid, phenytoin, carbamazepine, clobazam, lacosamide, lamotrigine, levetiracetam, topiramate, primidone, or phenobarbital	
(xvii) Active outpatient prescription for an anticoagulant medication with a significant metabolic interaction with phenobarbital (i.e., warfarin or apixaban)	
(xviii) Active outpatient prescription for a monoamine oxidase inhibitor (e.g., phenelzine, selegiline, tranylcypromine, isocarboxazid)	
(ixx) Renal failure, as defined by a creatinine clearance <10 ml/min (as calculated by the Cockcroft-Gault equation) and/or active receipt of renal replacement therapy (dialysis)	
(xx) Administration of IV or oral phenobarbital during the index admission prior to randomization.	

### Recruitment and consent

Patients will be recruited from the emergency department, wards, and intensive care units of Unity Health – St. Michael’s Hospital. St. Michael’s is an academic hospital with a focus on urban health located in Toronto, Canada. Informed consent will be obtained by appropriately trained research staff. We have obtained ethics approval to use a hybrid consent model wherein we will prioritize obtaining consent from the patient or his/her substitute-decision-maker (SDM) at the time of recruitment whenever possible. However, we recognize that many patients with sAAWS will lack decision-making capacity and some may be estranged from family. In this circumstance, we will use deferred consent [19]. As such, eligible patients will be randomized and consent will be sought as soon as possible after randomization [19]. Use of a hybrid consent model will directly address the high loss to follow-up that was experienced in a prior trial of barbiturates conducted by Rosenson and colleagues [16].

### Randomization

Patients will be randomized in a 2:1 ratio to either a single IV dose of phenobarbital or placebo in addition to symptom-triggered diazepam (CIWA protocol). This allocation was chosen based on funding constraints and our desire to maximize the clinical experience with phenobarbital in this 39-patient pilot trial. Central randomization will be conducted by the Research Pharmacy Department of Unity Health Toronto – St. Michael’s Hospital using a list of random treatment assignments created using the R package RandomiseR (R Foundation for Statistical Computing, Vienna, Austria).

### Intervention

As soon as possible after randomization, patients will receive either a single IV dose of phenobarbital [7.5 mg per kg of ideal body weight (IBW), prepared in 250 ml D5W; *n*=26] or placebo (250 ml D5W; *n*=13) which will be infused intravenously over 1 h (i.e., at an infusion rate of 4.16 ml/min). The study drug and placebo will be prepared by the Research Pharmacy and will be identical in appearance; all other trial participants will be blinded to treatment allocation. In an emergency, the patient’s attending physician will be able to request that the steering committee reveal which treatment they received. As ideal body weight is derived from patient height, the phenobarbital doses administered are anticipated to be in the range of 400–600 mg. A dose of 7.5 mg/kg was selected to enable both therapeutic benefit and minimize the risk for adverse events (e.g., respiratory depression and hypotension).

**Fig. 1:**
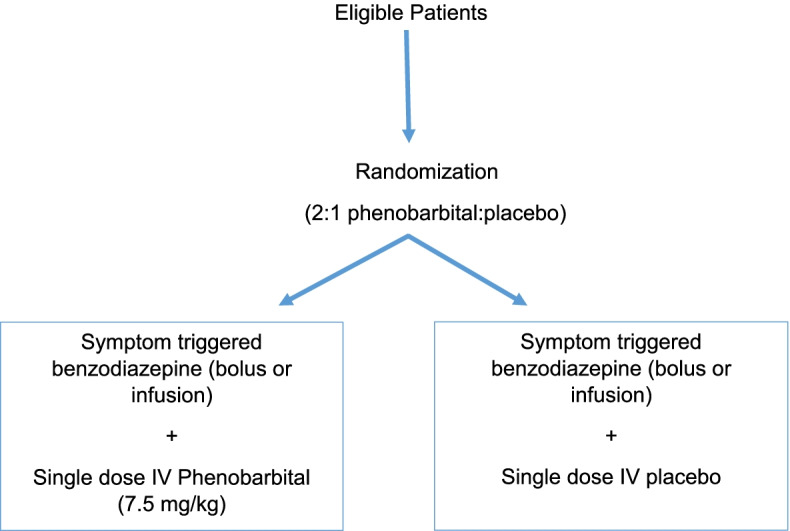
Trial design. IV, intravenous

During study drug administration, patients will be monitored with continuous oximetry, non-invasive blood pressure measurement every 30 min (+/− 5 min), and continuous cardiac monitoring in a high-acuity unit (e.g., ICU, cardiac intensive care unit, ED, step-up unit). Although phenobarbital achieves peak serum concentration within 1 h of administration, we will monitor study participants with continuous pulse oximetry and non-invasive blood pressure measurement every 30 min (+/− 5 min) for a minimum of 3 h after the start of study drug administration in this pilot trial. Each patient will therefore be monitored for 4 h (i.e., 1 h during study drug administration and 3 h after the start of study drug administration to ensure the safety of trial participants). There are no additional trial-mandated interventions. The day of study drug infusion will be considered study day 1. Patients will be followed daily for the following 6 days (i.e., until study day 7), or until the time of hospital discharge. Additional doses of phenobarbital will be prohibited for enrolled patients to avoid treatment contamination until after day 7 (Fig. 1).

### Data collection

Research personnel will maintain screening logs and collect (i) *patient demographics* (age, gender, housing, comorbidities, time since last alcohol consumption, baseline laboratory investigations, and CIWA-Ar scores [18]); (ii) *treatment:* number, dose, and type of benzodiazepine or alternative treatments administered (e.g., haloperidol, propofol), vital signs, use of non-invasive or invasive ventilation, need for monitored setting or bedside sitters over 7 days or until hospital discharge; and (iii) *feasibility and outcomes data.*

Initial data will include medication use prior to randomization, date and time of emergency room admission, data and time of hospital admission, current care environment, time and location of study drug administration, age at randomization, sex, housing status, ideal body weight, time since last drink, Charlson comorbidity score, serum sodium, creatinine, hemoglobin, white blood cell and platelet counts, pulse oximetry, heart rate, blood pressure, home use of non-invasive positive pressure ventilation, primary admitting diagnosis, and maximal initial CIWA-Ar score [18].

Daily data collection will include patient disposition, daily medication use, maximal CIWA-Ar score [18], and the occurrence of adverse events, if applicable. The completion of an Addictions Medicine consultation, the use of physical restraints, and the presence of a bedside sitter (where applicable) will be recorded. The elapsed time from randomization until a subject has three consecutive CIWA-Ar scores of 10 or less will be recorded [18]. Data pertaining to medication use will include cumulative use of oral lorazepam and diazepam, intravenous lorazepam, midazolam, and diazepam, and any use of alternative second-line treatments (e.g., propofol, clonidine, dexmedetomidine, haloperidol, carbamazepine, ketamine, baclofen, or additional phenobarbital). We will review patient charts and electronic databases to record clinical outcomes (length of stay in a monitored care setting after randomization, time to Addictions Medicine consultation, length of stay in hospital after randomization, vital status (alive/deceased) at hospital discharge). A REDCap database (with appropriate range-checks for data entry) will be used to maintain data integrity. Data security and confidentiality will be maintained as per Health Canada regulations. Patients who wish to withdraw from the pilot trial (e.g., following enrollment under deferred consent) will be asked whether we can prospectively follow them to include their outcomes. Unless they agree, their data will be excluded from the planned analyses and additional patients will be randomized in order to meet our target recruitment.

We will record adverse events including intubation, aspiration, seizure, use of non-invasive (NIV) or invasive ventilation. We will also document medication extravasation, systolic blood pressure (SBP) measurement below 90 mmHg, medication interactions, exacerbations of chronic disease (including myasthenia, porphyria, megaloblastic anemia), dermatologic reactions (i.e., Stevens-Johnson/Toxic Epidermal Necrolysis), and elevations in liver enzymes (toxic hepatitis). We will record cardiac arrest or peri-arrest events (i.e., Code Blue events) and the etiology of these events. Serious adverse events will be reviewed by the Data Safety and Monitoring Committee. Serious and unexpected adverse events will be reported to Health Canada and the research ethics board. The trial may be subject to an unplanned audit by Health Canada during its course. No provision has been made for compensation in the event that a trial subject should suffer harm.

### Outcomes

We will evaluate feasibility metrics in the PHENOMANAL pilot trial. The primary outcome of interest will be the ability to recruit 39 patients within 24 months at Unity Health Toronto – St. Michael’s Hospital. We will also assess clinicians’ ability to deliver the assigned treatment (i.e., the frequency of protocol violations). We will consider recruitment feasible if we can recruit 2–3 patients per month over the expected 24-month recruitment period. We will consider compliance rates > 80% to be acceptable in both study arms and a crossover rate of < 10% (from control to phenobarbital) to be acceptable. Preliminary data will be reported to summarize patient demographics, treatment efficacy and safety, and consent withdrawals.

### Statistical analysis

We will compare preliminary estimates of treatment effect between the alternative treatment strategies using the chi-squared test (alternatively, Fisher’s exact test) and Student’s *T* test (alternatively, Wilcoxon rank-sum test) for binary and continuous outcomes, respectively. We reasoned that a sample size of 39 patients would ensure that we could assess our feasibility metrics (recruitment, consent, protocol adherence) and that our multidisciplinary team members would gain experience with both protocols. Analyses will be performed in SPSS (SPSS Inc., Chicago, USA). In subgroup analyses, we will examine for differences in selected outcomes (e.g., length of stay in hospital, monitored care setting) separately for patients admitted to hospital more than 48 h prior to randomization vs. those randomized within 48 h.

### Trial oversight and monitoring

The trial will be conducted under the supervision of a Steering Committee, consisting of four physicians, as per the Steering Committee Terms of Reference. A Data Safety and Monitoring Board (DSMB), consisting of two physicians and a statistician, will monitor the trial and review serious adverse events.

Severe adverse events (hypotension below a systolic blood pressure of 90 mmHg, respiratory depression or distress requiring intubation, cardiac arrest or pre-arrest events (e.g., “Code Blue” events), and the etiology of these events) will be reviewed by the DSMB, who may recommend to the Steering Committee to stop the trial. Given that patients with severe AAWS are a high-risk population for adverse events, we will review and adjudicate which, if any events, are most likely the result of the study drug rather than the underlying pathology of alcohol withdrawal. Protocol amendments will be reviewed by the Research Ethics Board of St. Michael’s Hospital and by Health Canada prior to communication to all trial participants.

## Discussion

The PHENOMANAL pilot trial is one study within a *program of research* that includes a scoping review, cross-sectional survey [6], and the proposed pilot trial*.* In a multicenter, multidisciplinary survey of ED physicians, internists, intensivists, and psychiatrists, members of our group found that four medications (haloperidol, clonidine, phenobarbital, and propofol) were commonly used second-line treatments for sAAWS. Forty-two (40%) respondents already used phenobarbital in practice. Of these, 23 (55%) of respondents used phenobarbital in patients who were refractory to benzodiazepines, and most preferred to use IV (vs. oral) phenobarbital (66% vs. 29%, *p*<0.001) [6].

This pilot trial addresses a clinical dilemma and is relevant to the patient population served at inner-city hospitals. Patients with sAAWS face multiple healthcare and social inequities. In planning the trial, we encountered several challenges including difficulty securing funding and the need for multiple regulatory approvals and multidisciplinary engagement in protocol design and implementation. Although alcohol withdrawal is a common health problem, trials of treatment strategies for sAAWS fall into a funding void in Ontario. Since the investigation of novel treatments for patients with sAAWS is not entirely medical or psychiatric, studies evaluating new treatments fall outside the usual funding purview of many local and provincial funding agencies. In addition to securing research ethics approval, we obtained Health Canada approval to conduct this pilot trial. Health Canada approval was necessary as we will be administering phenobarbital for a novel indication in the PHENOMANAL trial. In addition, we secured a clinical trial exemption from Health Canada to administer phenobarbital as a controlled substance.

Alcohol use disorder is a stigmatized condition that is more common in marginalized patients. Patients with sAAWS also pose unique challenges in trial implementation with regard to consent and safety. We recognize that many patients with sAAWS lack decision-making capacity and many are estranged from family. Consequently, we plan to use a hybrid consent model wherein patient or substitute decision-maker (SDM) consent will be prioritized. However, for patients who lack decision-making capacity and those that do not have available SDMs, we will consider deferred consent [19]. With this model, after multiple documented attempts to contact SDMs, research personnel will enroll eligible patients. As soon as possible after randomization, written consent will be sought from patients or SDMs.

In designing and implementing the trial, we addressed challenges including the need to identify patients outside of regular research hours and safety issues related to patients who express a desire to leave against medical advice after receiving study intervention. To maximize our ability to identify and recruit eligible patients during the trial period, physicians will rotate weeks “on call” to identify eligible trial participants after hours and on weekends. To facilitate after hours enrollment, we will have a pharmacist on call to prepare and dispense study medication for the trial. Additionally, monthly fees, as well as call back fees, will enable after hours preparation of study drugs. For trial participants who refuse further participation or receipt of intervention and wish to leave the hospital after receiving phenobarbital or placebo, we will mandate a 6-h observation period. The responsible clinical team will assess the patients’ ability to provide self-care and the need for further observation. The DSMB will monitor the frequency of patients leaving against medical advice and all adverse events. Although studying patients with sAAWS presents several challenges, primary research is needed to advance care in this patient population.

The PHENOMANAL trial is novel in several ways. First, it investigates a new treatment for a potentially fatal and understudied condition that more commonly affects marginalized individuals. As such, it addresses an important evidence gap. Second, it seeks to repurpose an existing medication for a novel indication. Third, it seeks to advance methodology in acute care research through the use of a hybrid consent model and inform the design of a large-scale trial. Finally, the design and implementation of the PHENOMANAL trial has forged a novel collaboration involving clinicians from Critical Care, Emergency Medicine, Internal Medicine, Psychiatry (Addictions), and Clinical Pharmacy.

## Data Availability

Not applicable for the protocol manuscript. Data for the trial will be made available upon written request to the corresponding author.
